# Development of Candida auris Short Tandem Repeat Typing and Its Application to a Global Collection of Isolates

**DOI:** 10.1128/mBio.02971-19

**Published:** 2020-01-07

**Authors:** Theun de Groot, Ynze Puts, Indira Berrio, Anuradha Chowdhary, Jacques F. Meis

**Affiliations:** a Department of Medical Microbiology and Infectious Diseases, Canisius Wilhelmina Hospital (CWZ), Nijmegen, The Netherlands; b Centre of Expertise in Mycology Radboudumc/CWZ, Nijmegen, The Netherlands; c Medical and Experimental Mycology Group, Corporación para Investigaciones Biológicas (CIB), Medellín, Colombia; d Hospital General de Medellin Luz Castro de Gutiérrez ESE, Medellín, Colombia; e Department of Medical Mycology, Vallabhbhai Patel Chest Institute, University of Delhi, Delhi, India; f Department of Medical Microbiology, Radboudumc, Nijmegen, The Netherlands; Duke University

**Keywords:** *Candida auris*, genotyping, infection control, short tandem repeats

## Abstract

Candida auris is a pathogenic yeast that causes invasive infections with high mortality. Infections most often occur in intensive care units of health care facilities. It is crucial to trace the source and prevent further spread of C. auris during an outbreak setting; therefore, genotyping of C. auris is required. To enable fast and cost-effective genotyping, we developed a short tandem repeat (STR) typing assay for C. auris. STRs in C. auris were identified, and from an initial selection of 23 STRs, 12 were used to develop a STR typing assay. Having shown that the STR typing assay was reproducible and specific, a robust set of 444 C. auris isolates was investigated to identify genotypic diversity. In concordance with whole-genome sequencing (WGS) analysis, we identified five major different C. auris clusters of South American, South Asian, African, East Asian, and Iranian origin. Overall, a total of 40 distinct genotypes were identified, with the largest variety in the South Asian clade. Comparison with WGS demonstrated that isolates with <20 single nucleotide polymorphisms (SNPs) are mostly not differentiated by STR analysis, while isolates with 30 or more SNPs usually have differences in one or more STR markers. Altogether, a highly reproducible and specific STR typing assay for C. auris was developed; this assay distinguishes the five different C. auris clades in identical fashion to WGS, while most isolates differing by >30 SNPs, as determined via WGS, are also separated. This new C. auris-specific genotyping technique is a rapid, reliable, and cost-effective alternative to WGS analysis to investigate outbreaks.

## Abstract (precis)

Candida auris is an emerging fungal pathogen now recognized as a threat to public health. The pathogen has spread worldwide and causes mainly hospital-associated outbreaks. To track and trace outbreaks and to relate them to new introductions from elsewhere, whole-genome sequencing and amplified fragment length polymorphism (AFLP) have been used for molecular typing. Whole-genome sequencing is costly and available only at a few centers, and AFLP is a complicated technique and hard to interpret. We describe a novel simple STR genotyping technique based on short tandem repeats in the C. auris genome. We also show that the performance of this STR-based genotyping technique has proven comparable to that of WGS. Overall, this work provides a novel, rapid, reliable, and cost-effective method of molecular outbreak investigations of C. auris.

## INTRODUCTION

Candida auris is a pathogenic yeast that was first isolated in South Korea in 1996 and first reported in 2009, when it was isolated from a Japanese patient who had a C. auris infection of the external ear canal (1, 2). In the following decade, this yeast species was found in locations all over the world (3–8), including South Africa, India, Pakistan, Kuwait, Venezuela, United Kingdom, and United States, suggesting that its emergence might be a consequence of climate change (9). Fungemia and wound infections are the most common clinical conditions caused by C. auris, which could subsequently lead to dissemination (10). The mortality rate in patients with C. auris infections, who often experience severe disease, reaches levels of 30 to 60% (10, 11). A serious complication in the treatment of patients is the resistance of C. auris to multiple antifungal agents, such as fluconazole and amphotericin B (12). Most C. auris isolates are sensitive to echinocandins, although around 5% of isolates are reported to be resistant to this class of antifungals (12).

Besides its elevated pathogenicity and multidrug resistance, C. auris is also highly transmissible. It colonizes the nose, axilla, and groin and is frequently found on inanimate surfaces and reusable equipment in health care facilities, which are potential sources of transmission among hospitalized patients (13–15). It is challenging to clean contaminated surfaces and equipment, as C. auris can form biofilms that are relatively insensitive to hydrogen peroxide and chlorhexidine (16). Due to its high degree of infectivity and relative insensitivity to standard cleaning protocols, C. auris has caused outbreaks in various health care institutions, especially in intensive care settings (13).

In 2017, whole-genome sequencing (WGS) demonstrated the existence of four different C. auris clades (17). These specific geographical clades were identified as the South Asian, South American, African, and East Asian clades. Subsequently, WGS study of C. auris isolates from various hospitals within the United States demonstrated the presence of all four genetically diverse clades, suggesting that U.S. patients became colonized or infected with isolates from three continents (18). This spread of C. auris in the United States demonstrates that travel and/or migration plays an important role in spreading this disease. The identification of C. auris in a routine microbiology laboratory is difficult. As it is often misidentified as other *Candida* spp., like C. haemulonii, C. sake, C. famata, C. lusitaniae, and C. parapsilosis or as Cryptococcus laurentii or Rhodotorula glutinis, the exact burden of C. auris outbreaks remains unknown and challenging to determine (19–21). A specific, rapid, accurate, reproducible, and easy typing method is essential to determine the presence of a potential outbreak; however, so far such a method is not yet available for C. auris. In this study, we developed a short tandem repeat (STR)-based typing for C. auris and used it to type a global collection of isolates.

## RESULTS

### Selection of STR markers.

Tandem repeats in the haploid C. auris genome were identified using genomic information for four isolates originating from different clades. Then 23 tandem repeats of 2, 3, 6, or 9 nucleotides were selected; these tandem repeats shared at least three repeats with an identical unit length. In order to map the 200 to 300 bases flanking the tandem repeats on both sides, primers were designed (see Table S1 in the supplemental material) and applied in PCR amplification using 10 isolates from four known clades. PCR products were found for 22 tandem repeats, and these were sequenced. One of the tandem repeats was not present in all clades, while the flanking sequences of five tandem repeats harbored deletions/insertions close to the tandem repeat, making them unsuitable for STR analysis (Table S1). After excluding two repeats that showed very little variability in copy number between the isolates (Table S1), a total of 14 tandem repeats remained. As these tandem repeats included only two tandem repeats with a length of six nucleotides, these were also excluded, leaving three dinucleotide, six trinucleotide and three nonanucleotide repeats.

10.1128/mBio.02971-19.2 TABLE S1 Overview of STR selection and primers. Download Table S1, DOCX file, 0.02 MB. Copyright © 2020 de Groot et al. 2020 de Groot et al. This content is distributed under the terms of the Creative Commons Attribution 4.0 International license.

### Development of C. auris STR typing assay and its application to a global collection of isolates.

**FIG 1:**
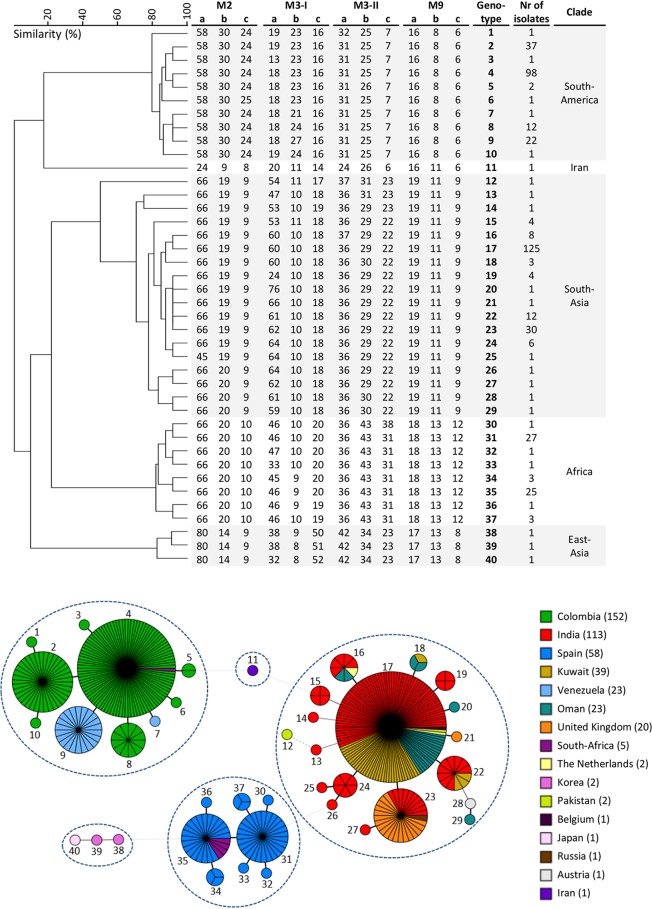
STR genotypes and minimum spanning tree of 444 C. auris isolates. UPGMA dendrogram of STR genotypes (top panel) and mimimum spanning tree (bottom panel) of 444 C. auris isolates originating from various countries. (Top) Cluster analysis showed that the different clades form distinct clusters based on the STR profiles, demarcated in the dendrogram by the gray background. Nr, number. (Bottom) The branch lengths in the minimum spanning tree (MST) indicate the similarity between isolates with thick solid lines (variation in one marker), thin solid line (variation in two markers), thin dashed lines (variation in three markers), and thin dotted lines (variation in more than eight markers). The numbering in the MST indicates the genotype numbers, while the number of isolates per country are shown in the color key.

To develop a STR typing assay for C. auris, primers were designed in close proximity to the tandem repeat. After these primers were tested and optimized, they were coupled to fluorescent probes. The four multiplex PCRs (M2, M3-I, M3-II, and M9) were then used to genotype a C. auris collection of 444 isolates from 16 different countries. Most of these isolates originated from different hospital outbreaks in South America, Europe, and South Asia. All isolates were successfully typed using these multiplex panels, with the exception of the three isolates from South Korea and Japan, which required monoplex typing of the M3-I panel. Most repeats, with the exception of the nonanucleotide repeats, demonstrated stutter peaks, due to established PCR artifacts (22). An overview of repeat characteristics, number of alleles, and Simpson index of diversity (*D*), which ranged from 0.58 to 0.82, is shown in Table 1. Among 444 C. auris isolates, 40 different genotypes containing 1 to 125 isolates were identified (Fig. 1A). The genotypes clustered in five different groups, previously identified via WGS as the five different C. auris clades (17, 23). These five clades were differentiated by at least 8 to 10 STR markers. Less variation was found within the different clades, as the maximal number of different STR markers between isolates within the South Asian clade was seven, while within the other C. auris clades, excluding Iran, isolates differed in maximally three STR markers. The total number of different alleles for the three markers in the M2 panel was five, while there were, respectively, six or seven and three or four alleles in the M3-II and M9 panel. M3-Ib and M3-Ic exhibited, respectively, 8 and 9 alleles, while there were 20 alleles for STR marker M3-Ia. To visualize the genotypes and the country of origin, all individual isolates are shown in a minimum spanning tree (Fig. 1B), which demonstrated that some genotypes were found in different countries. Interestingly, one of the South African isolates (MOL353) localized in the South American clade. Whole-genome sequencing confirmed the overlap of this isolate with South American isolates (NCBI accession number SRX6733158).

**TABLE 1:** Overview of PCR primers for selected STR loci, concentration used in multiplex PCR, details of repeat characteristics, discriminatory index, and genomic site

PCR panel and primer name	Primer sequence (5′–3′)		Concn^*b*^ (pmol/μl)	No. of bases of primer- flanking sequence	Repeat unit	No. of repeats^*c*^			No. of alleles	*D* value^*d*^	Intragenic/ locus of the protein-coding gene^*e*^
	Forward primer^*a*^	Reverse primer				Min	Max	Ref			
M2											
M2a	FAM-GCAACATCCTGAGCAGTATCAC	GGTGTTGACGTGCCCAAATATGC	8	168	AG	24	80	66	5	0.58	Intragenic
M2b	JOE-CCACTCCGTTTTGGGTCTG	AGAGAATCTACAAATGTGTCGC	3	67	AG	9	30	19	5	0.69	Intragenic
M2c	TAMRA-CTGTTTCTGTGGCAGGCTTCC	GCCACGTTTCACYGCYACCAT	2	90	AG	8	25	9	5	0.68	Intragenic

M3-I											
M3-Ia	FAM-GCATGGATCAACAGCTAACAG	AGTGCCAGGCTGTGTACTTTTG	8	124	CAA	13	76	60	20	0.82	Intragenic
M3-Ib	JOE-CATCCTAACGCTGGCTCTTC	GGYTTTGAGGYTGCCCTAGC	3	145	CAA	8	27	10	8	0.69	CJJ09_004096
M3-Ic	TAMRA-GCAACTACGCATTGTGTATTC	CTAACAGAGGATTTCAATTGCC	3	124	TTA	14	52	18	9	0.69	Intragenic

M3-II											
M3-IIa	FAM-GTTCAAAATCGCTGACGGTC	GAGATGATGATGGCACTTGC	8	101	CTA	24	42	36	6	0.60	CJJ09_003318
M3-IIb	JOE-GTGAATGGAGCACCACAACCAG	GCGCAAATGACTGGCCCATG	3	155	GTA	25	43	29	7	0.70	CJJ09_002311
M3-IIc	TAMRA-GTGATGAGCGCACTACACAGG	GGCGAAGAAACGGTGAGTAC	2	79	CAA	6	38	22	6	0.69	Intragenic

M9											
M9a	FAM-CTTGTCTAGTTTGCGATCTACGC	GAGACTGCCAAGCCAAGC	8	127	GATGATGAA	16	19	19	4	0.68	CJJ09_001802
M9b	JOE-CTGCTTACTGGAGACTCTTCC	GATGAGGAGGACGAGGACG	4	104	TCATCGTCA	8	13	11	3	0.68	CJJ09_000617
M9c	TAMRA-GTACGAAATGGGGATAATTGGG	ACCAACCGTGCTATTCTC	2	110	TCCTTCTTC	6	12	9	4	0.68	CJJ09_002457

a FAM, 6-carboxyfluorescein; JOE, 4',5'-dichloro-2',7'-dimethoxy-fluorescein; TAMRA, 6-carboxytetramethylrhodamine.

b The concentrations for the forward and reverse primers are the same.

c Min, minimum; Max, maximum; Ref, reference strain. The reference strain, with genotype 17, is CDC388 (B11098).

d Discriminatory power of STR assay as determined via the Simpson index of diversity.

e Locus according to strain B11245.

### Reproducibility, stability, and specificity of C. auris STR.

To test reproducibility, isolates GMR-OM028 and VPCI 213/P/15 were independently amplified five times in four replicate experiments. STR typing demonstrated identical results for both isolates for all STR markers, demonstrating that the method is highly reproducible. The stability of the STR markers was tested by subcloning 20 colonies of two strains for 5 generations and analyze the STR markers. The copy number was not altered in any of the STR markers (data not shown). In order to determine the specificity of our STR assay for C. auris, we analyzed 15 other yeast species for all 12 markers. Products were found only with C. duobushaemulonii using the M3-IIb and M3-IIc marker and with *C. pseudohaemulonii* using the M2c marker, demonstrating that in general, the markers are highly specific for C. auris.

## DISCUSSION

The present study describes the development of a novel C. auris STR genotyping analysis. This C. auris-specific STR assay consists of four multiplex PCRs, which amplify 12 STR targets with a repeat size of 2, 3, or 9 nucleotides (panels M2, M3-I, M3-II, and M9). The assay appeared reproducible and specific, while its markers remained stable after subculturing for more than 100 generations. STR genotyping was performed on 444 C. auris isolates from various geographical regions, which yielded highly concordant results with WGS and five similar distinct groups, corresponding with the four well-known clades from South America, Africa, South Asia, and East Asia, and the possible new clade from Iran (17, 23, 24). The allelic variations in the 444 isolates resulted in 40 different genotypes. This relatively low number of different genotypes is also reflected by the relatively low *D* values of the different STR markers and is partly due to the fact that >95% of our isolates originated from hospital outbreaks, leading to the inclusion of many clonal strains. Furthermore, it is known that C. auris only recently emerged and that there is still little variation between isolates from the same clade, as also shown by WGS (18). Although the ability to discriminate between isolates would be in general better with WGS, the cutoff to determine relatedness between isolates remains to be established for both methods. The current study is subject to limitations. The stability testing as done by subculturing does not necessarily represent its stability in an outbreak situation. Thus, further research is needed to determine the stability of STR markers in an outbreak and the use of this assay to determine the relatedness between more closely related isolates.

### Large overlap in typing data obtained with the STR assay and WGS analysis.

In order to obtain more insight in the utility of this new STR assay to type strains in an outbreak of C. auris, we compared its results with strains previously analyzed by WGS performed by the Centers for Disease Control and Prevention (CDC) (17, 18). In the present study, we typed 25 isolates, obtained from India, Pakistan, South Africa, and Venezuela by STR which were previously analyzed using WGS (see Fig. S1 in the supplemental material) (17). WGS demonstrated four clades, differentiated by at least tens of thousands of SNPs, while via STR analysis, these four clades differed by at least eight markers. Within the South American clade, the isolates from Venezuela (*n* = 5) were differentiated by a maximum of 17 SNPs, while STR profiles from these isolates were identical. A total of four isolates from South Africa analyzed by both methods were differentiated by a maximum of 11 SNPs by WGS and did not show any difference by STR analysis. From the South Asian clade, 14 out of 15 isolates were differentiated by ∼60 SNPs by WGS, while with STR analysis, these isolates demonstrated variations in markers M3-Ia and M3-Ib (genotypes 15 and 17 and genotypes 22 to 24). Out of these 14 isolates, there were 5 and 7 isolates with identical STR data (genotypes 17 and 23) with maximally 55 and 29 SNPs, respectively. Interestingly, two of these isolates (isolate B11209 with genotype 15 and isolate B11214 with genotype 23), which originated from Indian hospitals in Kochi in 2013 and New Delhi in 2014, were found to be identical with WGS (18), while STR analysis demonstrated differences in two STR markers. This discrepancy might be due to the elimination of repetitive DNA sequences from most WGS SNP analyses, as the high degree of variability complicates the SNP counts. The 15th isolate, B8411, harbored ∼800 SNPs compared to the other 13 isolates (17, 18) which corresponded with differences in 6 STR markers (genotype 12). Interestingly, Chow et al. demonstrated with WGS that the Japanese isolate JCM 15448 differed in 34 and 35 SNPs compared to the two isolates KCTC17809 and KCTC17810 from South Korea, while STR typing demonstrated differences in two or three markers between the Japanese and South Korean isolates (18). The South Korean isolates were differentiated by 19 SNPs with WGS and one copy number in two STR markers. Altogether, isolates that differed by a few SNPs (<20) via WGS and are labeled as almost indistinguishable are often also not differentiated with STR analysis, while most isolates that differed in 30 or more SNPs are differentiated by STR analysis in one or more STR markers.

10.1128/mBio.02971-19.1 FIG S1 Comparison of genotypic differences of C. auris isolates via STR and WGS analysis. Lockhart et al. (17) provided detailed information regarding the precise number of SNPs differentiating 47 C. auris isolates via WGS (left panel), which was compared with the STR genotypes of isolates that were included in the present study (right panel). The maximal differences in SNPs between isolates with genotypes 23, 17, 35, and 9 were, respectively, 29, 55, 11, and 17. Gt, genotype. Download FIG S1, PDF file, 0.6 MB. Copyright © 2020 de Groot et al. 2020 de Groot et al. This content is distributed under the terms of the Creative Commons Attribution 4.0 International license.

### Identifying the relatedness of isolates is potentially feasible using STR typing.

Implementation of a typing method in an outbreak setting requires establishing cutoff values to determine the potential relatedness of isolates. As the mutation rates in microorganisms strongly differ, such cutoff values should be determined for each microorganism separately (25). To establish a cutoff value to determine the relatedness between isolates, we analyzed the variation between several hospital outbreaks included in this study. In the 2015–2016 outbreak in London, United Kingdom, all isolates but one exhibited a single genotype (26). The difference of four copy numbers was observed in marker M3-Ia, suggesting that small variations (copy number of <5) in STR marker M3-Ia may not be used to regard strains as nonrelated. Analysis of the outbreak in a Spanish hospital (27) showed eight different genotypes with differences in the M3-Ia marker, although differences in three other M3 markers were also found. All genotypes in the Spanish outbreak localized in the African clade. Due to nonavailability of WGS and epidemiological information of Spanish isolates, it is not possible to understand whether this population was possibly genetically heterogeneous at its point of introduction, although the larger number of genotypes within one outbreak makes this very likely. From the outbreaks in Colombia, we found that isolates originating from hospitals in Santa Marta, Cartagena, and Medellin all exhibited genotype 4. Most isolates from the outbreaks in Popayan and Bogota exhibited a single genotype (genotype 2 and 4, respectively), although in both outbreaks there were a few single isolates with other genotypes, caused by one copy number in one marker. Finally, most isolates from Barranquilla, which originated from one hospital and were isolated between April 2015 and January 2019, clustered in two larger groups, while a few isolates exhibited a different genotype. Also, these isolates differed only in one copy number of one marker with the exception of isolate C72900 (isolated 28 December 2018), which exhibited 13 repeats for M3-I, while the other isolates had 18 or 19 repeats. Thus, the variation between the Colombian isolates was minimal, with the exception of isolate C72900, found at the very end of the outbreak, which might be an independent introduction. Altogether, the variation between isolates within the same clade is relatively small, as also indicated by the relatively low *D* values for the different STR markers. As a consequence, it will be challenging to identify potential relatedness between isolates found within a hospital, when these isolates belong to the same clade. On the basis of the current data, we suggest that small variations (copy number <5) in STR marker M3-Ia or one copy number in a M2 or other M3 STR marker should likely not be used to label strains as nonrelated, while more copy number variations in these STR markers or variation in a M9 STR marker strongly indicates that isolates are not related. To establish a reliable cutoff for relatedness for the C. auris STR, more STR data with its concomitant epidemiological data and WGS analysis is required.

In summary, we developed a STR typing assay for C. auris that is reliable, reproducible, and specific. While C. auris typing via WGS analysis will ultimately lead to the most accurate discrimination regarding the relatedness of isolates, STR typing has the advantage that it is less expensive, faster, and easier. As such, this STR assay will allow many labs to type C. auris during an outbreak.

## MATERIALS AND METHODS

### Isolates.

For the short tandem repeat (STR) analysis, 444 C. auris isolates from Austria (28), Belgium (29), Colombia (hospitals in Barranquilla, Bogota, Cartagena, Medellin, Popayan, and Santa Maria), India (30), Iran (31), Japan (30), South Korea (30), Kuwait (24), Oman (32), Pakistan (17), Russia (33), South Africa (30), Spain (27), The Netherlands (34), United Kingdom (35), and Venezuela (6) were used. For a complete overview of isolates, see Table S2 in the supplemental material. Isolates were stored at −80°C according to standard procedures. Species identification via sequencing and/or matrix-assisted laser desorption ionization−time of flight mass spectrometry (MALDI-TOF MS) was conducted as described previously (30).

10.1128/mBio.02971-19.3 TABLE S2 Overview of isolates, identification numbers, and country/city of origin. Download Table S2, DOCX file, 0.04 MB. Copyright © 2020 de Groot et al. 2020 de Groot et al. This content is distributed under the terms of the Creative Commons Attribution 4.0 International license.

The specificity of the STR was tested on a spectrum of yeast isolates, including those that are previously known to yield misidentification of C. auris by commercial biochemical methods. The following isolates were tested: C. haemulonii CBS5149T, CBS7802, CBS7801, and CBS5150; C. pseudohaemulonii JCM12453T, KCTC1787, and CBS10004T; C. duobushaemulonii CBS7796, CBS7800, CBS7799, and CBS9754; C. tropicalis ATCC 750; C. dubliniensis CBS8500; C. albicans ATCC 10231; C. glaebosa clinical isolate; C. krusei ATCC 6258; C. glabrata ATCC 2175; C. lusitaniae Canisius Wilhelmina Hospital (CWZ) identification number (ID) 10-11-02-05; C. parapsilosis CWZ ID 10-06-05-86; C. sake clinical isolate; Cryptococcus gattii CBS12652; Cryptococcus albidus clinical isolate; and Rhodotorula glutinis CWZ ID 10-06-05-66.

### Identification of STR loci.

Genome scaffolds of isolates B8441-Pakistan, B11220-Japan, B11221-South-Africa, and B11243-Venezuela were downloaded from NCBI (17). Scaffolds from each isolate were combined in one fasta file (www.bioinformatics.org/sms2/combine_fasta.html), and this fasta file was uploaded in tandem repeat finder (http://tandem.bu.edu/trf/trf.html; 36) using the basic search option. From the resulting list of STRs, those repeats that contained insertions or deletions, exhibited <90% perfect match of the repeat sequence, did not vary in copy number between the isolates, or contained repeat sequences within the potential PCR primer regions were excluded.

### Primer design, PCR, and genotyping.

Primers were designed using the Tm calculator and Multiple Primer Analyzer from ThermoFisher Scientific, ordered via Eurogentec (Cologne, Germany). The PCR for amplification of the STR flanking regions was performed on a Thermocycler (Westburg, Biometra, Göttingen, Germany) using 1× Fast Start *Taq* polymerase buffer with MgCl_2_, 0.2 mM deoxynucleoside triphosphates (dNTPs), 25 pmol forward (fwd) and (rev) primer, 1 U Faststart *Taq* polymerase (Roche Diagnostics, Germany), water, and DNA. A similar setup was used for the multiplex PCRs with 4.5 to 20 pmol fwd or rev primer with the following thermal protocol: 10 min of denaturation at 95°C, followed by 30 cycles, with 1 cycle consisting of 30 s of denaturation at 95°C, 30 s of annealing at 60°C, and 1 min of extension at 72°C, and a final incubation for 10 min at 72°C. For DNA sequencing, the product was purified according to the Ampliclean method (NimaGen, Nijmegen, The Netherlands), and the sequencing PCR was performed using 0.5 μl BrilliantDye premix, 1.75 μl BrilliantDye 5× sequencing buffer (NimaGen), 5 pmol fwd or rev primer, 5.75 μl water, and 1 μl DNA. After D-Pure purification (NimaGen) sequencing was performed using the 3500XL genetic analyzer (Applied Biosystems, Foster City, CA, USA) and analyzed in Bionumerics 7.6.1 (Applied Maths, Kortrijk, Belgium). For the STR analysis, samples were diluted 1:1,000 and 10 μl of the diluent, together with 0.12 μl of Orange 600 DNA size standard (NimaGen), boiled for 1 min at 95°C, and analyzed according to the manufacturer’s recommendations on an automatic sequencer, ABI 3500XL genetic analyzer (Applied Biosystems).

### Whole-genome sequencing.

Genomic libraries were prepared and sequenced with Illumina technology (Illumina, San Diego, CA, USA) with 2 × 150-bp paired-end read mode at Eurofins Genomics (Ebersberg, Germany). Seventy-four C. auris whole-genome sequencing (WGS) sequences from NCBI were added to the analysis. FastQC and PRINSEQ were used to assess the quality of read data and perform read filtering. Read data were aligned against a publicly available genome sequenced on PacBio RS II using BWA. Single nucleotide polymorphism (SNP) variants were identified using SAMtools and filtered using the publicly available SNP analysis pipeline NASP to remove positions that had less than 10× coverage, less than 90% variant allele calls, or that were identified by Nucmer as being within duplicated regions in the reference. Phylogenetic analysis and bootstrapping with 1,000 iterations were performed on SNP matrices using RAxML.

### Culture and DNA isolation.

Isolates were grown on Sabouraud agar plates at 35°C. To test the stability of STR markers, 20 colonies of isolates 10-08-01-01 and VPCI 247/P/15 were clonally expanded for 5 generations on Sabouraud agar plates. For DNA sequencing, strains were resuspended in a vial with 400 μl MagNA Pure Bacteria lysis buffer and MagNA Lyser green beads and mechanically lysed for 30 s at 6,500 rpm using the MagNA Lyser (all Roche Diagnostics GmbH, Mannheim, Germany). Subsequently, DNA was extracted and purified with the MagNA Pure LC instrument and the MagNA Pure DNA isolation kit III (Roche Diagnostics), according to the recommendations of the manufacturer. For STR analysis, strains were resuspended in 50 μl physiological salt, and after the addition of 200 U of lyticase (Sigma-Aldrich, St. Louis, MO, USA) and incubation for 5 min at 37°C, 450 μl physiological salt was added. The sample was then incubated for 15 min at 100°C and cooled down to room temperature.

### Data analysis and discriminatory power.

The copy numbers of the 12 markers of all isolates were determined using GeneMapper software 5 (Applied Biosystems). The size of the alleles was rounded. Relatedness between isolates was analyzed using BioNumerics software version 7.6.1 (Applied Maths) via the unweighted pair group method with arithmetic averages (UPGMA) using the multistate categorical similarity coefficient. All markers were given an equal weight. The discriminatory power of the STR assay was determined using the Simpson index of diversity (*D*) as described previously (22). A *D* value of 1.0 indicates that according to the typing method used, all isolates have a different genotype, while a *D* value of 0 indicates that all isolates are identical.

### Data availability.

The *Candida auris* WGS sequences were deposited in NCBI under accession no. SRX6733158.
